# Supplementary material for: Structural insights into the molecular effects of the anthelmintics monepantel and betaine on the Caenorhabditis elegans acetylcholine receptor ACR-23
Source: EMBO J. 2024 Jul 15;43(17):3787–806. doi: 10.1038/s44318-024-00165-7 (PMC11377560; doi:10.1038/s44318-024-00165-7)
Supplement: Supplementary file 1 — Appendix [file 44318_2024_165_MOESM1_ESM.pdf]

Appendix

Structural insights into the molecular effects of the anthelmintics monepantel and betaine on the *Caenorhabditis elegans* acetylcholine receptor ACR-23

Table of contents:

Appendix Fig. S1 2
Appendix Fig. S2 3
Appendix Fig. S3 4
Appendix Fig. S4 5
Appendix Fig. S5 6
Appendix Fig. S6 7
Appendix Fig. S7 8
Appendix Fig. S8 9
Appendix Fig. S9 10
Appendix Fig. S10 11
Appendix Fig. S11 12
Appendix Fig. S12 13
Appendix Fig. S13 14
Appendix Fig. S14 15
Appendix Table S1 16
Appendix Table S2 17

Appendix Figures and Legends

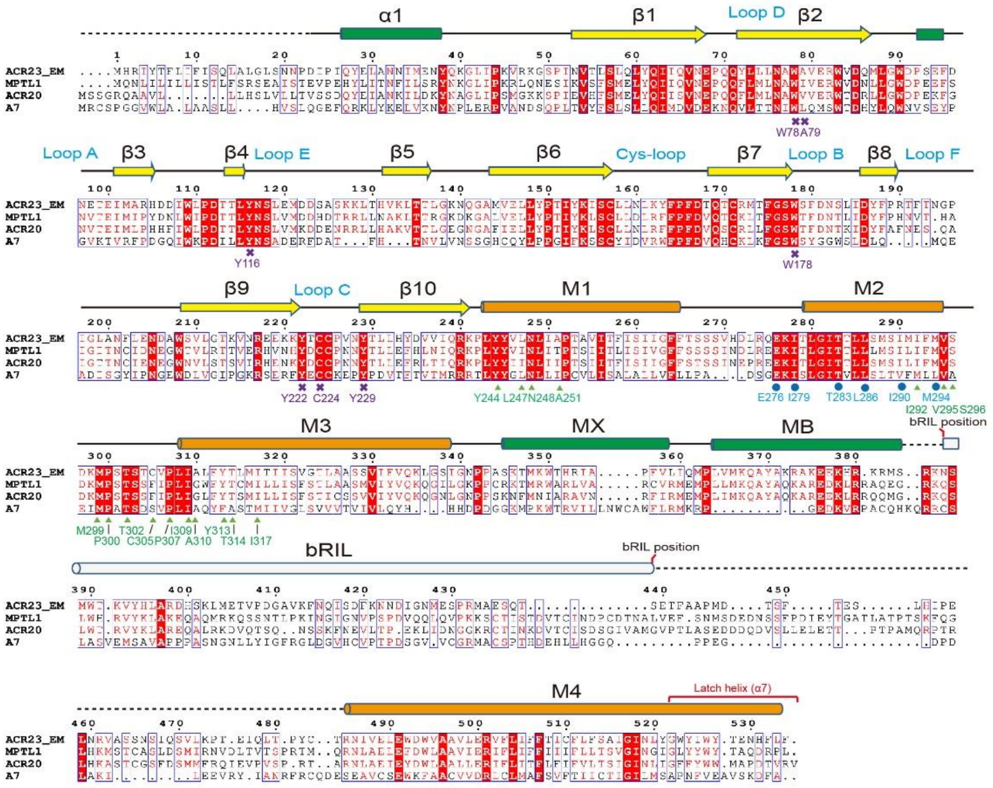

Appendix Fig. S1. Sequence alignment of ACR-23 and other DEG3 family member, as well as α7 nicotinic acetylcholine receptor.

Sequence alignment of ACR-23 (accession number NP_001317822.1), MPTL1 (accession number ART34953.1), ACR-20 (accession number NP_001367183.1), and α7 nicotinic acetylcholine receptor (accession number NP_000737.1). Secondary structures are indicated by cylinders (helices), arrows (β-strands), solid line (loop), and dashed line (disordered region). The replacement location for bRIL is indicated by a white cylinder. Loops involved in neurotransmitter binding are labeled in blue. Conserved residues are highlighted in red. Purple crosses mark residues involved in betaine binding. Green triangles mark residues involved in monepantel binding. Blue circles mark pore-lining residues. Multiple sequence alignment was performed using Clustal Omega (https://www.ebi.ac.uk/Tools/msa/clustalo/), and this figure is prepared using ESPript server (https://espript.ibcp.fr/ESPript/ESPript/)

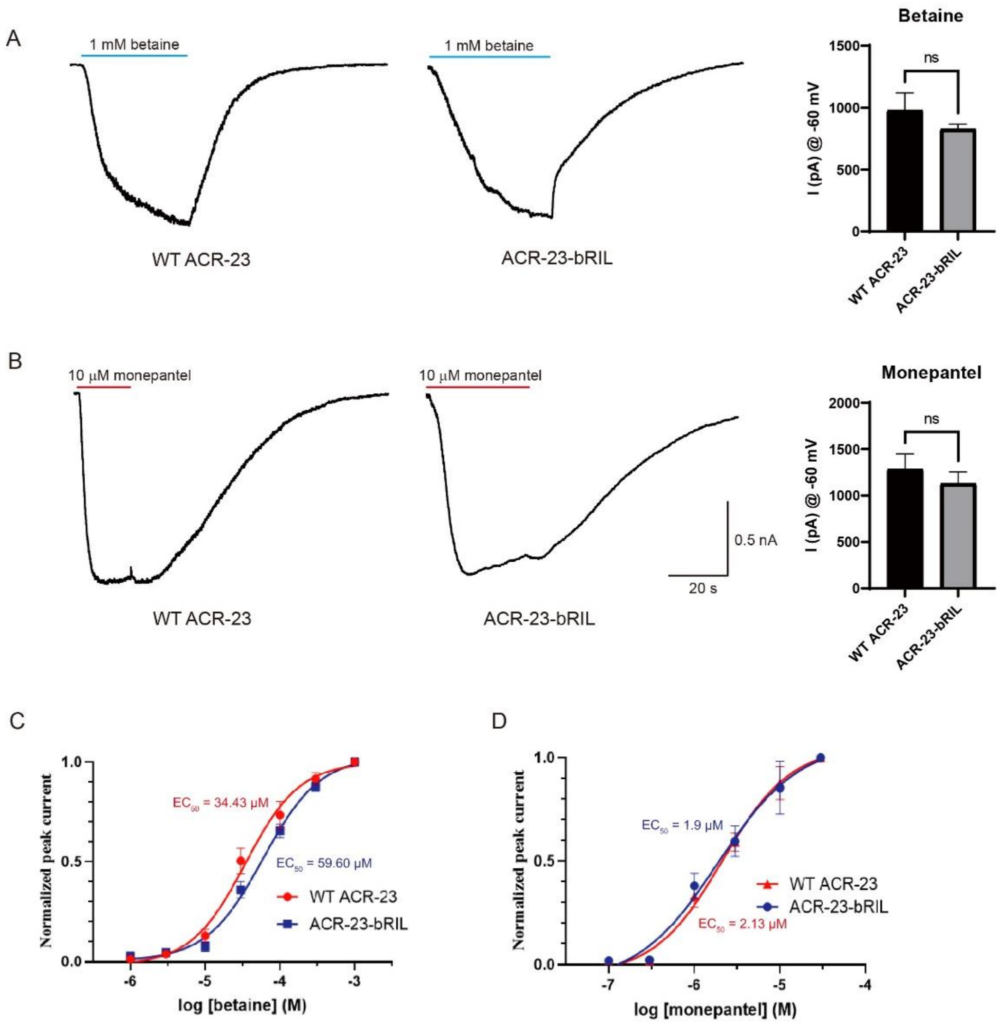

Appendix Fig. S2. Whole-cell patch-clamp electrophysiology of WT ACR-23 versus the EM construct. (**A-B**) Representative whole-cell current of WT ACR-23 and ACR-23-bril (EM construct) evoked by (**A**) 1 mM betaine (*P* value is 0.3762) or (**B**) 10 µM monepantel (*P* value is 0.4922). The statistics of current amplitude evoked by the agonists are shown on the right. (**C-D**) Dose-dependent activation of WT ACR-23 and ACR-23-bRIL by betaine (**C**) and monepantel (**D**). Data represent the mean±s.e.m. Statistical significance was determined using unpaired t-test with Welch's correction. ns indicates not significant.

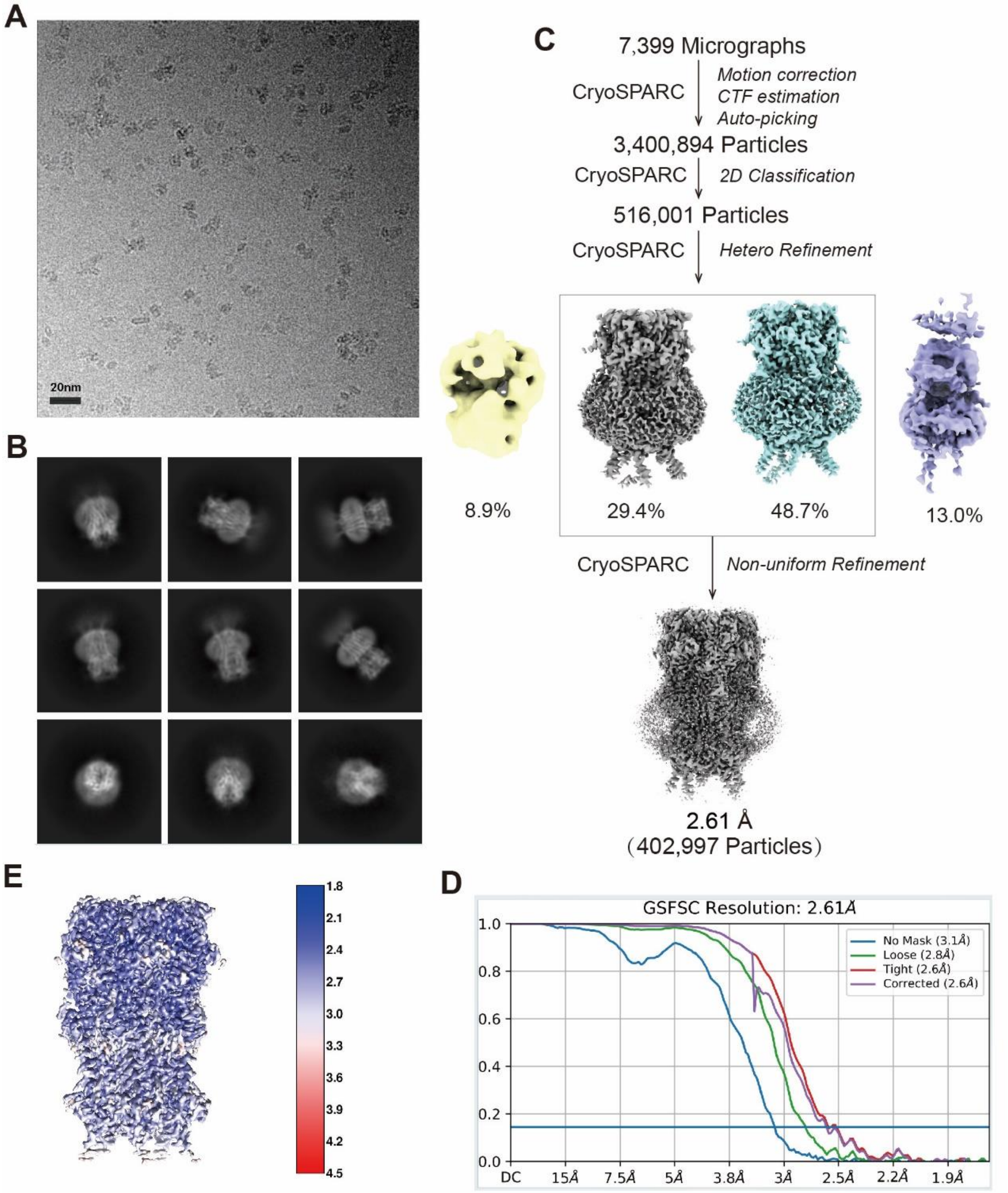

Appendix Fig. S3. Cryo-EM analysis of apo ACR-23. (**A**) A representative cryo-EM micrograph of apo ACR-23. Scale bar, 20 nm. (**B**) 2D class average images of ACR-23. (**C**) A brief workflow of cryo-EM image processing and reconstruction. (**D**) The GSFSC curve for the reconstruction. (**E**) Local resolution distribution for the density map of ACR23.

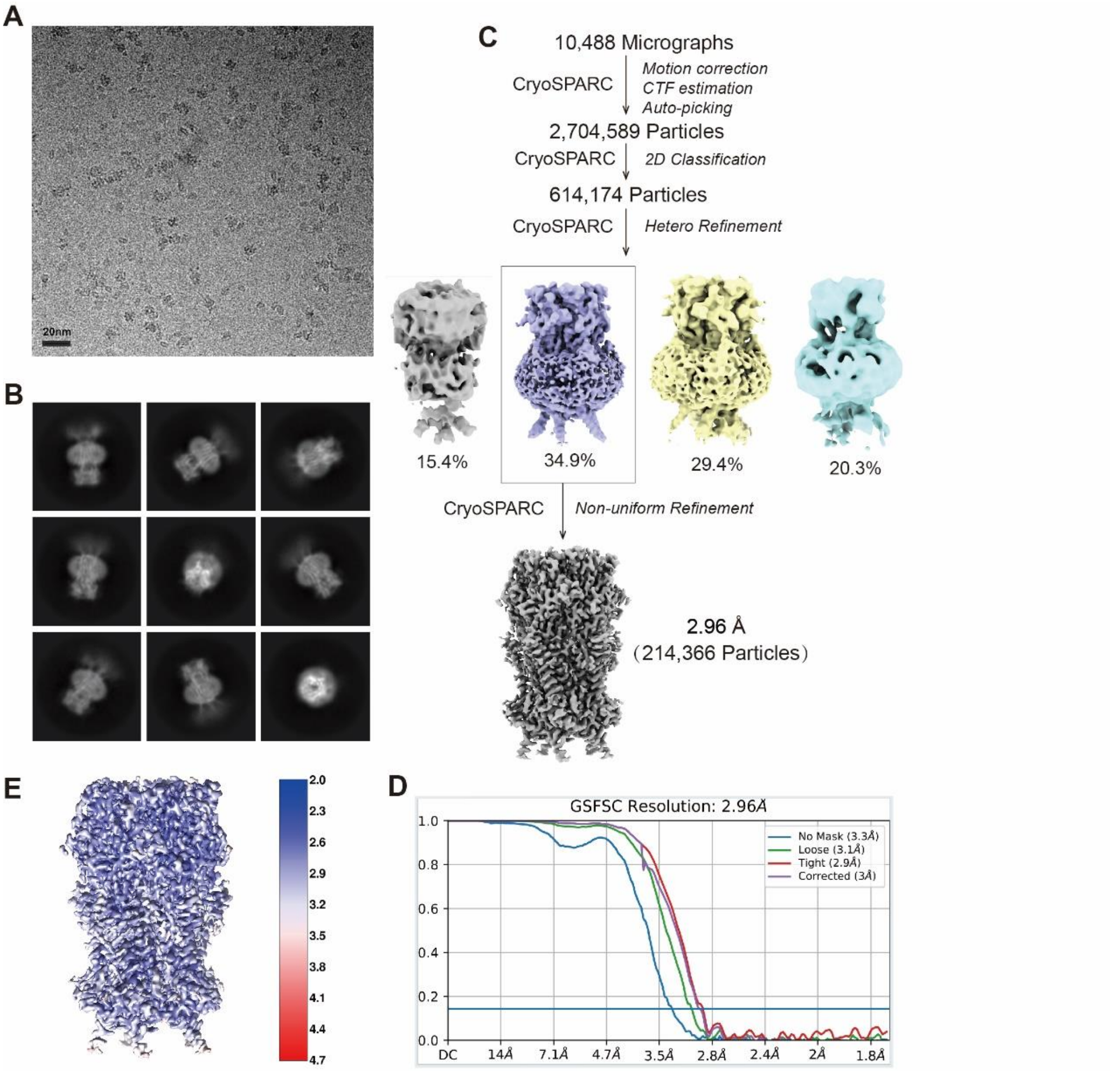

Appendix Fig. S4. Cryo-EM analysis of betaine-bound ACR-23. (**A**) A representative cryo-EM micrograph of betaine-bound ACR-23. Scale bar, 20 nm. (**B**) 2D class average images of betaine-bound ACR-23. (**C**) A brief workflow of cryo-EM image processing and reconstruction. (**D**) The GSFSC curve for the reconstruction. (**E**) Local resolution distribution for the density map of betaine-bound ACR-23.

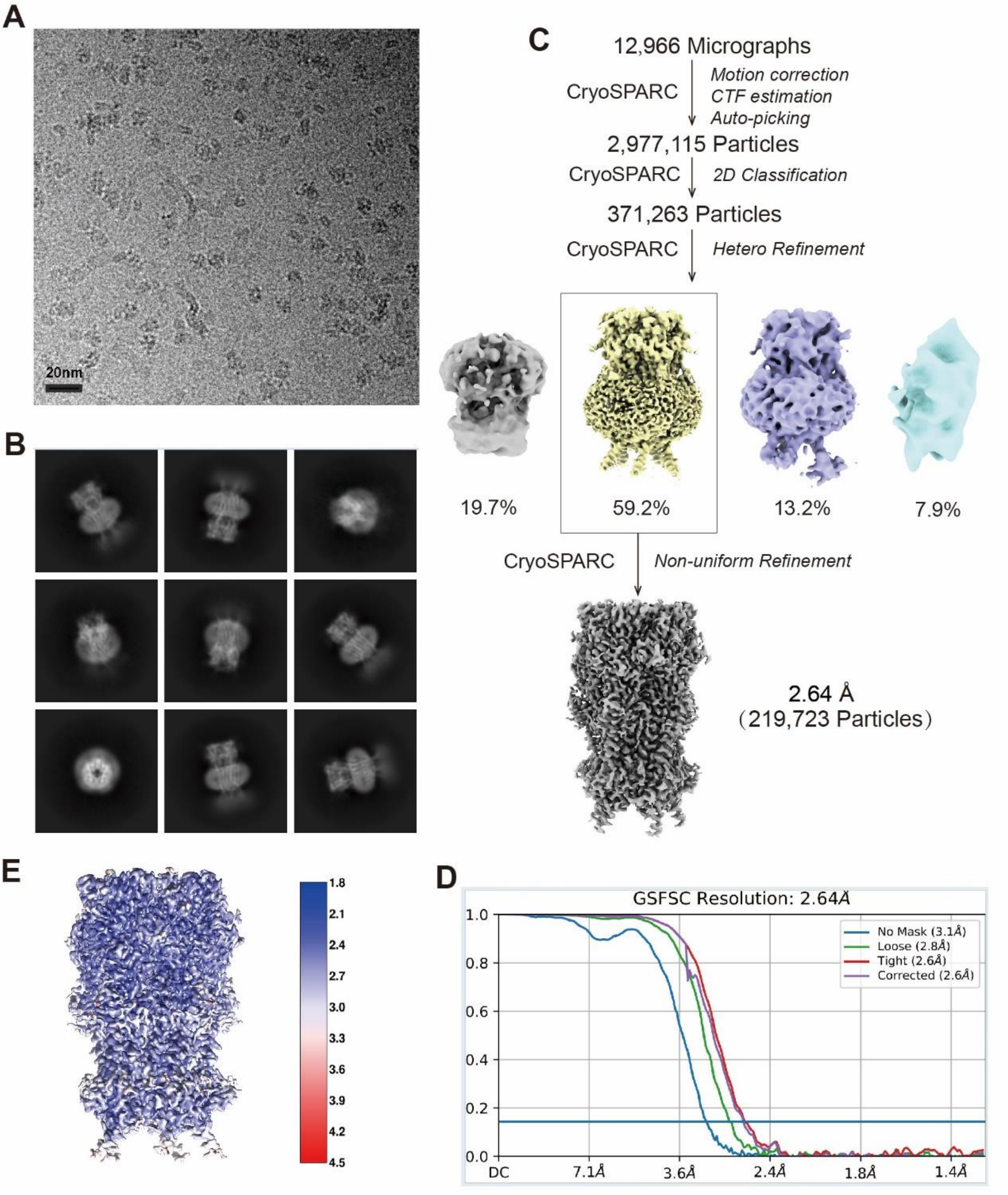

Appendix Fig. S5. Cryo-EM analysis of betaine&monepantel-bound ACR-23. (**A**) A representative cryo-EM micrograph of betaine&monepantel-bound ACR-23. Scale bar, 20 nm. (**B**) 2D class average images of betaine&monepantel-bound ACR-23. (**C**) A brief workflow of cryo-EM image processing and reconstruction. (**D**) The GSFSC curve for the reconstruction. (**E**) Local resolution distribution for the density map of betaine&monepantel-bound ACR-23.

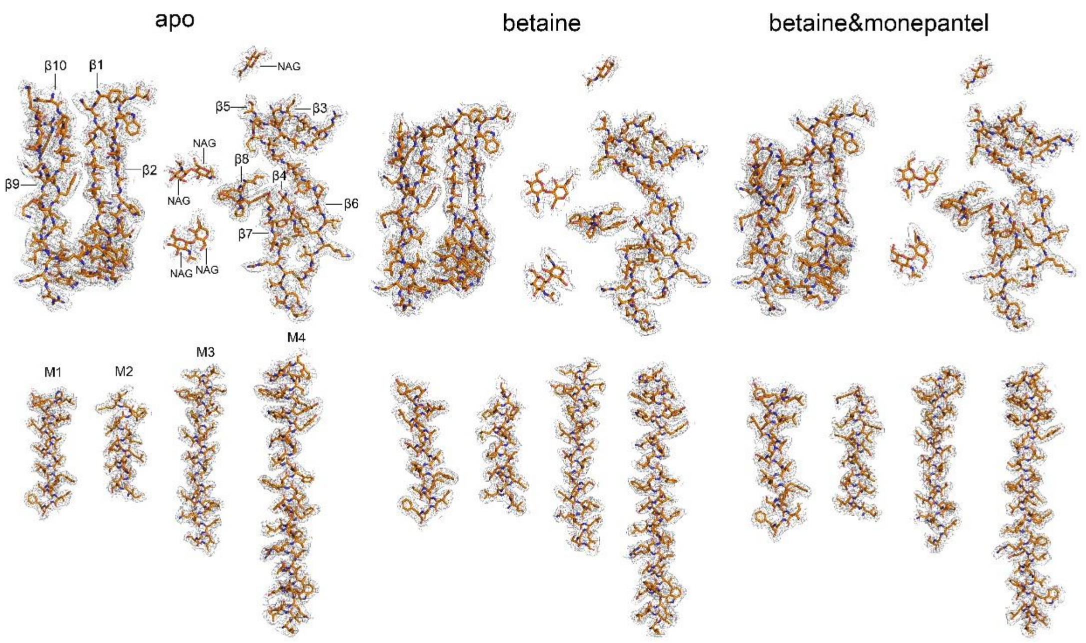

Appendix Fig. S6. Sample cryo-EM density maps for various parts (upper, ECD; bottom, TMD) of ACR-23 structures in different states (indicated on top). The maps are low-pass filtered to 3Å and sharpened with a temperature factor of −100 $Å^2$.

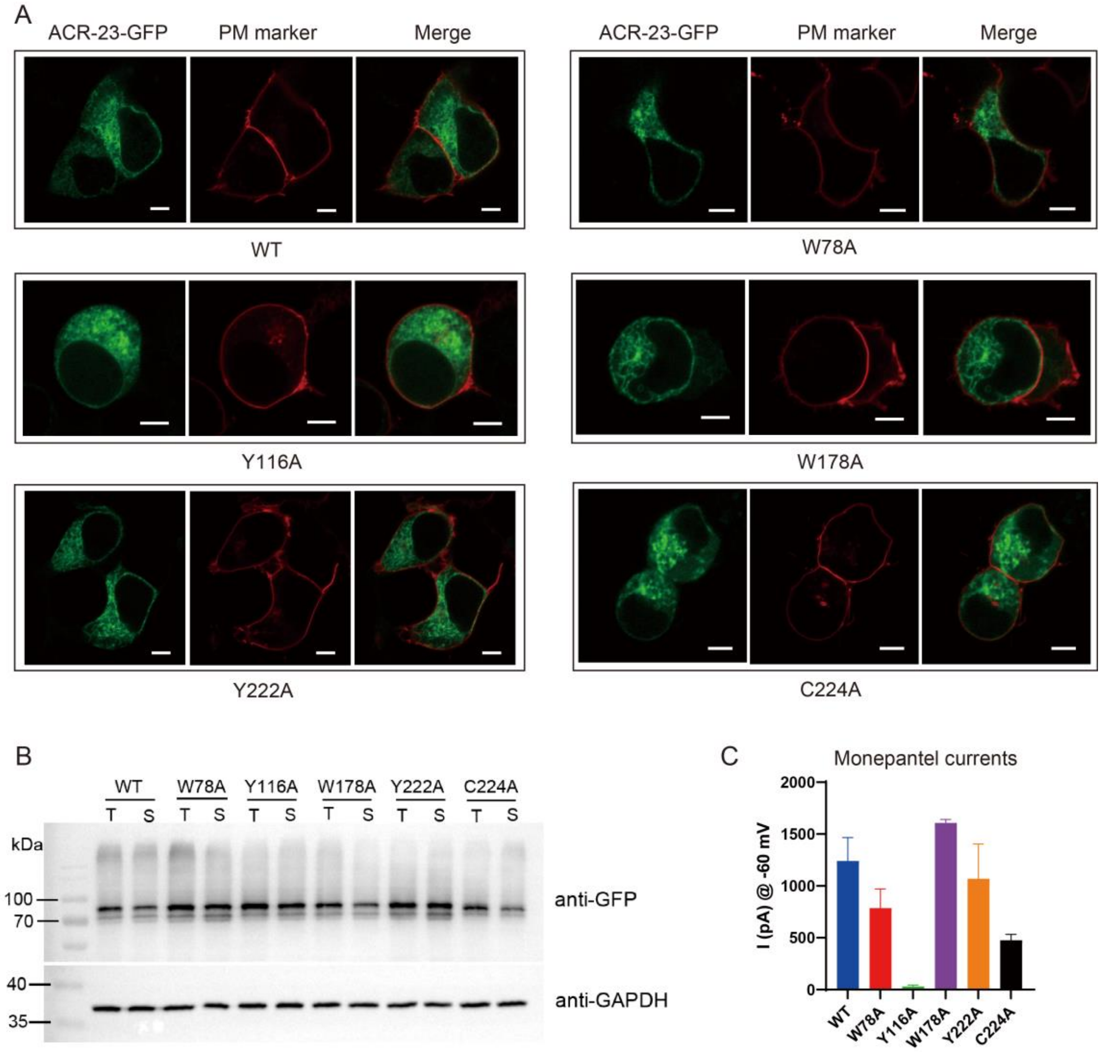

Appendix Fig. S7. Subcellular localization (A), protein expression (B) of betaine-binding site mutations and their activation by monepantel (C). (**A**) Subcellular localization as determined by confocal imaging. From left to right, three images in each box represent the indicated ACR-23 variant fused with GFP, membrane marker KRas GTPase fused with mCherry, and merge, respectively. The scale bars represent 5 μm. The experiment was repeated twice independently with similar results. (**B**) Protein expression level as determined by Western blotting. T: total extract after detergent solubilization. S: supernatant after centrifugation. The samples were detected by GFP and GAPDH (internal standard) antibodies. The experiment was repeated twice independently with similar results. In (**C**), data are peak amplitude of the currents for various ACR-23 mutants evoked by 10 μM monepantel and are presented as mean ± s.e.m. (biological replicates $n \geq 3$).

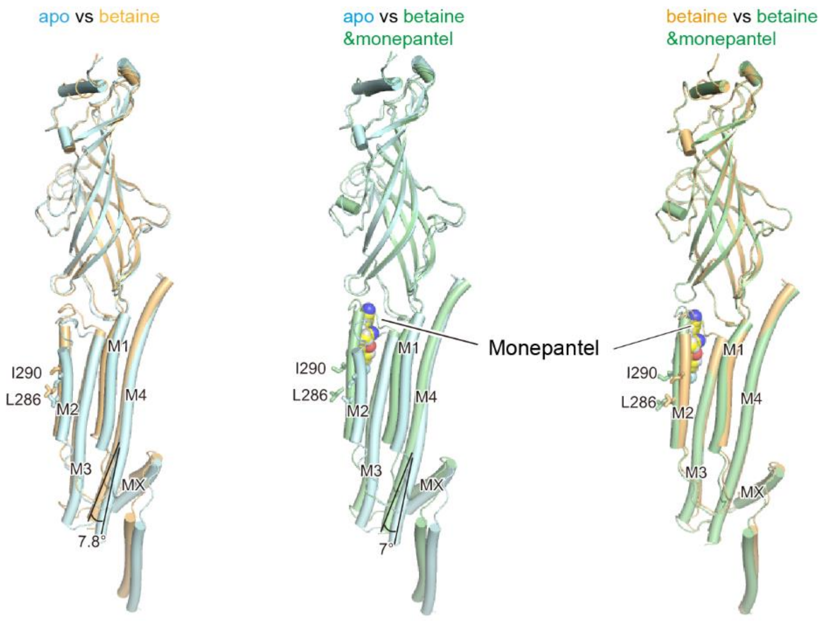

Appendix Fig. S8. Comparison of single subunit ACR-23 in apo, betaine-bound and betaine&monepantel-bound states. The ECD (22-241) is used for superposition. Key pore-lining residues are shown as sticks. The angle between M4 of each state is indicated. Bound monepantels are shown as spheres.

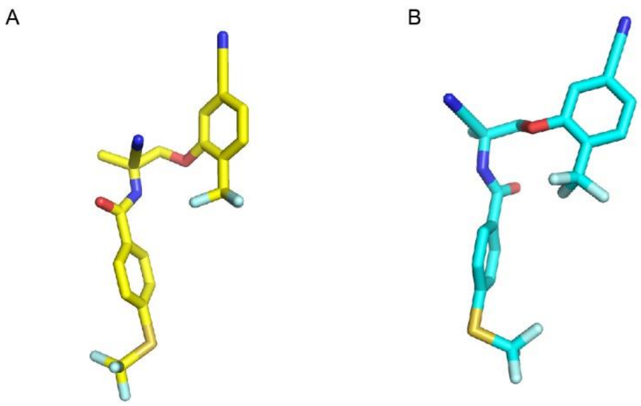

Appendix Fig. S9. Comparison of ACR-23 bound monepantel structure (A) and crystal structure of monepantel alone (B).

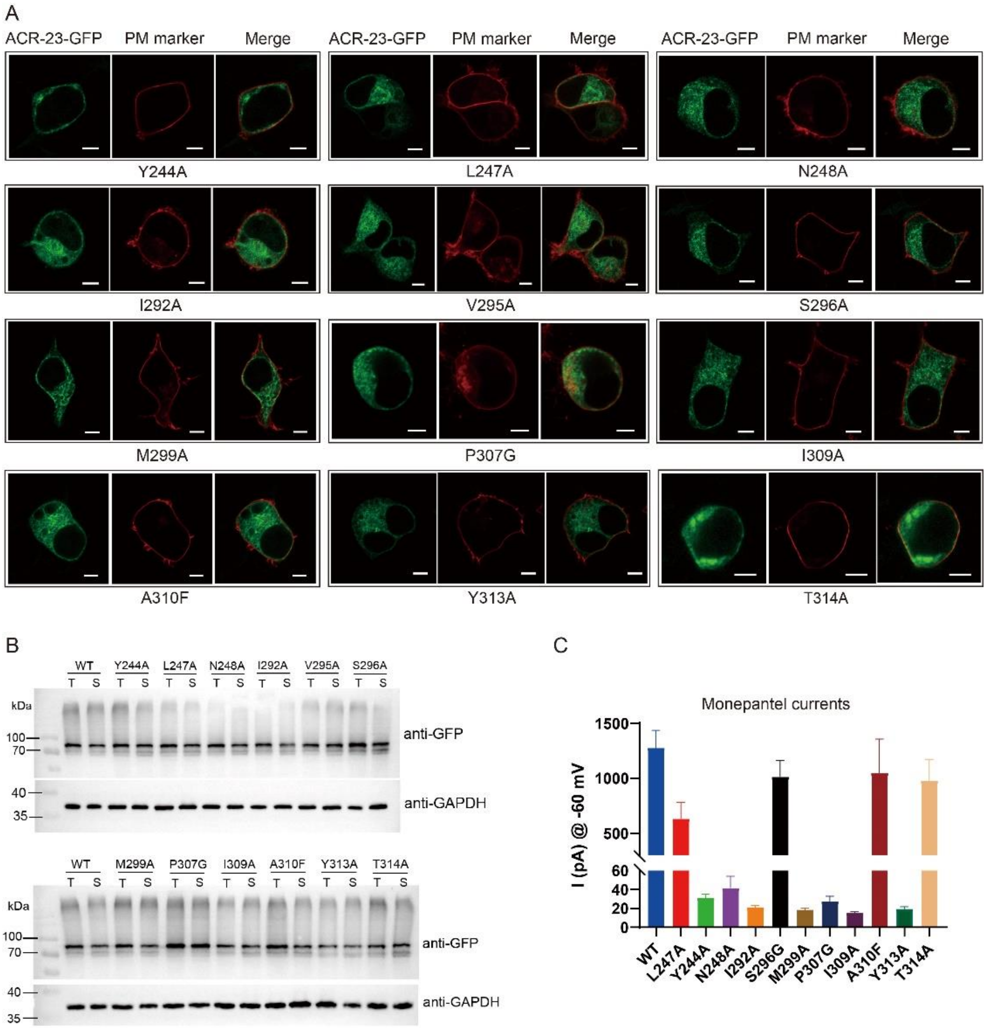

Appendix Fig. S10. Subcellular localization (A), protein expression (B) of monepantel-binding site mutations and their activation by betaine (C). (**A**) Subcellular localization as determined by confocal imaging. From left to right, three images in each box represent the indicated ACR-23 variant fused with GFP, membrane marker KRas GTPase fused with mCherry, and merge, respectively. The scale bars represent 5 μm. The experiment was repeated twice independently with similar results. (**B**) Protein expression level as determined by Western blotting. T: total extract after detergent solubilization. S: supernatant after centrifugation. The samples were detected by GFP and GAPDH (internal standard) antibodies. The experiment was repeated twice independently with similar results. In (**C**), data are peak amplitude of the currents for various ACR-23 mutants evoked by 1 mM betaine and are presented as mean ± s.e.m. (biological replicates n ≥ 3).

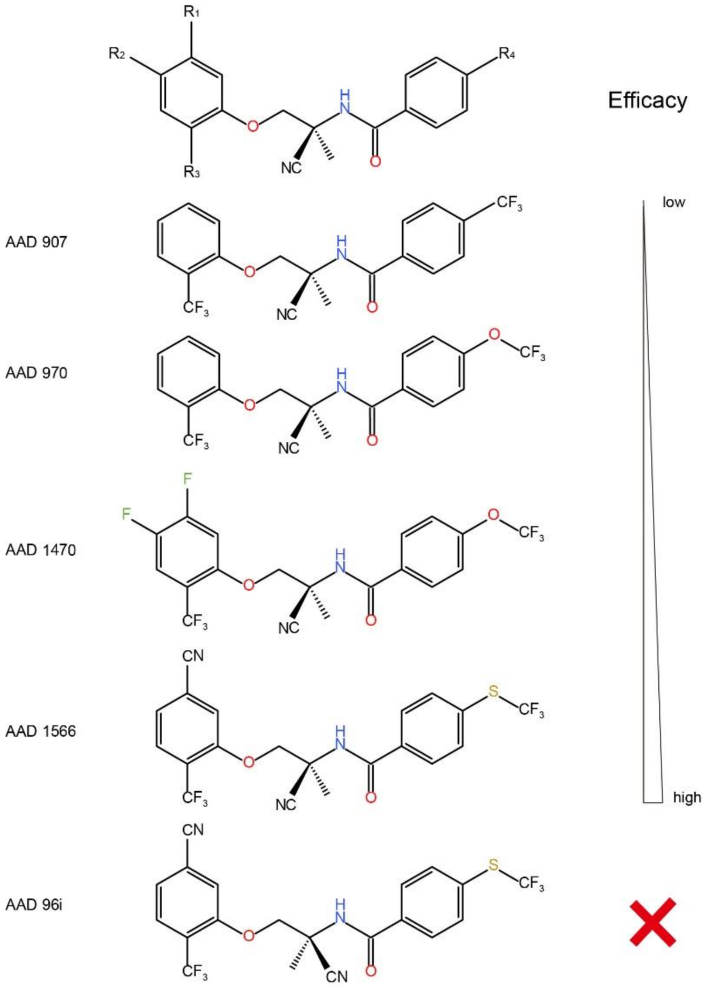

Appendix Fig. S11. Chemical structures of representative AADs. On the right, the triangle indicates the change in efficacy of different AADs as reported previously. AAD 96i, the other enantiomer in the same racemic as monepantel, which is inactive, is indicated by a red cross.

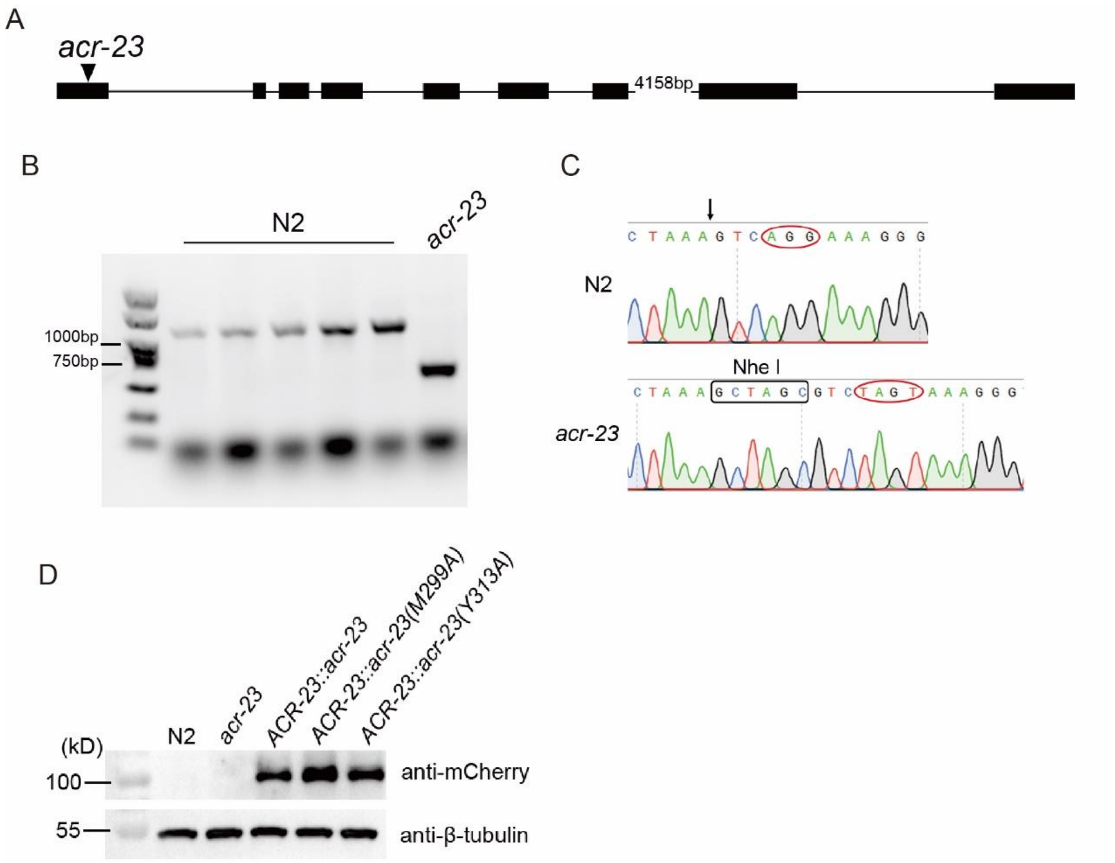

Appendix Fig. S12. Generation of *ACR-23* knockout strain (*acr-23*) (A-C) and detection of protein expression in the *c. elegans* strains used in this study (D). (A) Schematic representation of *acr-23* strain generation, with the position of premature stop codon indicated by black triangle. The black rectangles and the lines represent exons and introns respectively. (**B**) Verification of *acr-23* strain generation by PCR amplification and *Nhe*I digestion. **(C),** Sequencing analysis of *acr-23* strain. N2 is the wild type. (**D**) Protein expression level as determined by Western blotting. The samples were detected by GFP and β-tubulin (internal standard) antibodies. The experiment was repeated twice independently with similar results.

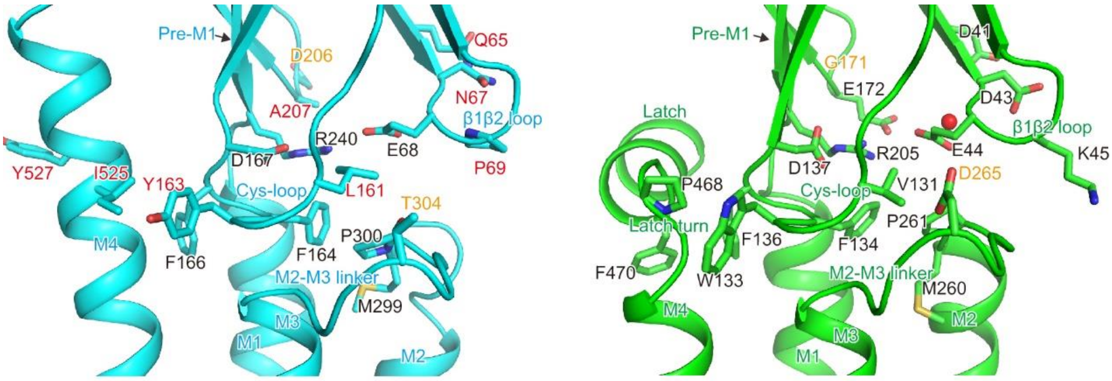

ACR23 (apo) α7 (apo, pdb# 7koo)

Appendix Fig. S13. Comparison of the ECD–TMD coupling region between ACR-23 and the α7 nicotinic acetylcholine receptor. Main chains are shown as cartoon and key residues are shown as sticks. Residues that are different from α7 nicotinic acetylcholine receptor are labeled in red, whereas residues involved in forming inter-subunit hydrogen bonds in ACR-23 and their counterparts in α7 nicotinic acetylcholine receptor are labeled in orange. Note that residues involved in forming a Ca^{2+} (red sphere) binding site in the α7 nicotinic acetylcholine receptor are mostly non-conserved except Glu 68.

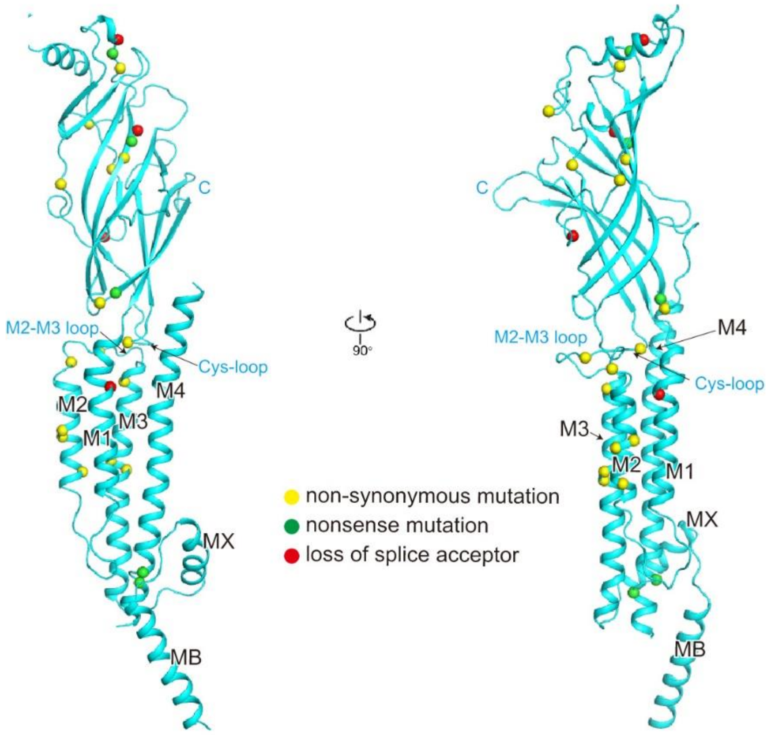

Appendix Fig. S14. Mapping of monepantel resistant mutations on ACR-23 structure. ACR23 is shown as cyan cartoon, and residues involved in different type of mutations are shown as spheres and colored differently as indicated.

Appendix Table S1. Data collection and refinement statistics

	ACR-23 Apo state (EMDB-60064) (PDB 8ZFL)	ACR-23 betaine-bound state (EMDB-60065) (PDB 8ZFM)	ACR-23 betaine& monepantel-bound state (EMDB-60063) (PDB 8ZFK)
**Data collection and processing**			
Voltage (kV)	300	300	300
Electron exposure (e–/Å^2)	~ 50	~ 50	~ 50
Defocus range (μm)	-1.2 ~ -2.4	-1.2 ~ -2.4	-1.2 ~ -2.4
Pixel size (Å)	0.84	0.84	0.84
Symmetry imposed	*C5*	*C5*	*C5*
Initial particle images (no.)	3,400,894	2,704,589	2,977,115
Final particle images (no.)	402,991	214,366	219,723
Map resolution (Å)	2.61	2.96	2.64
FSC threshold	0.143	0.143	0.143
**Refinement**			
Initial model used	*AF-G5EG88-F1*	*Apo model*	*Apo model*
Model resolution (Å)	2.61	2.96	2.64
FSC threshold	0.143	0.143	0.143
Map sharpening *B* factor (Å^2)	-80	-80	-80
Model composition			
Non-hydrogen atoms	17065	17115	17340
Protein residues	2060	2060	2060
Ligands	25	30	35
B factors (Å^2)			
Protein	55.99	54.16	45.95
Ligand	100.21	86.66	66.57
R.m.s. deviations			
Bond lengths (Å)	0.002	0.003	0.002
Bond angles (°)	0.474	0.542	0.453
Validation			
MolProbity score	1.1	1.78	1.82
Clashscore	3.12	7.65	13.56
Poor rotamers (%)	0.8	1.97	1.12
Ramachandran plot			
Favored (%)	98.04	97.6	97.21
Allowed (%)	1.96	2.4	2.79
Disallowed (%)	0	0	0

Appendix Table S2. Primers used in this study

Purpose	Primer name	Sequence
Protein expression for Cryo-EM analysis	ACR-23-FNhel	AAAAAAGCTAGCATGCACAGGATCTACACATTT
	ACR-23-RXhol	AAAAAACTCGAGAAAAAGAAAATGATTTTCCGTATA
	ACR-23-bril-5-F	GGAAACGGATGTCGAGAAAGATGGCCGAAGCAGGAG CAATGGCAGATTTGGAGGATAAC
	ACR-23-bril-5-R	GTTATCCTCCAAATCTGCCATTGCTCCTGCTTCGGCC ATCTTTCTCGACATCCGTTTCC
	ACR-23-bril-3-F	CCTACATCCAAAAGTACCTGTCACAAACATCCGAAACCTT
	ACR-23-bril-3-R	AAGGTTTCGGATGTTTGTGACAGGTACTTTTGGATGTAGG
Transient expression for Electrophysiology	ACR-23- FBglII	GTCCGGCCGGACTCAAGATCTCATGCACAGGATCTACACATTT
	ACR-23- RSacII	CGGTGGATCCCGGGCCCGCGGTCAAAAAAGAAAATGATTTTCG
Site directed mutagenesis	ACR-23-W78A-F	TATCTACTACTCAATGCG GCAGCGGTAGAGCGATGGGTA
	ACR-23-W78A-R	TACCCATCGCTCTACCGCTGCCGCATTGAGTAGTAGATA
	ACR-23-Y116A-F	CTGCCGGACACCACTTTG GCAAACTCACTTGAAATGGAT
	ACR-23-Y116A-R	ATCCATTTCAAGTGAGTTTGCCAAAGTGGTGTCCGGCAG
	ACR-23-W178A-F	CGAATGACATTTGGTAGT GCATCCTTTGACAATAGTCTA
	ACR-23-W178A-R	TAGACTATTGTCAAAGGATGCACTACCAAATGTCATTCG
	ACR-23-Y222A-F	AACCGCGAAGAGAAAAAA GCAACGTGCTGTCCGGTCAAC
	ACR-23-Y222A-R	GTTGACCGGACAGCACGTTGCTTTTTTCTCTTCGCGGTT
	ACR-23-C224A-F	GAAGAGAAAAAATACACGGCATGTCCGGTCAACTACACA
	ACR-23-C224A-R	TGTGTAGTTGACCGGACATGCCGTGTATTTTTTCTCTTC
	ACR-23-Y244A-F	ATTCAACGCAAACCGCTCGCATACGTGCTCAATCTGATC
	ACR-23-Y244A-R	GATCAGATTGAGCACGTATGCGAGCGGTTTGCGTTGAAT
	ACR-23-L247A-F	AAACCGCTCTACTACGTGGCAAATCTGATCGCGCCGACC
	ACR-23-L247A-R	GGTCGGCGCGATCAGATTTGCCACGTAGTAGAGCGGTTT
	ACR-23-N248A-F	CCGCTCTACTACGTGCTCGCACTGATCGCGCCGACCGCC
	ACR-23-N248A-R	GGCGGTCGGCGCGATCAGTGCGAGCACGTAGTAGAGCGG
	ACR-23-I292A-F	CTTTCGATGTCTATTATGGCATTTATGGTGTCCGACAAA
	ACR-23-I292A-R	TTTGTCGGACACCATAAATGCCATAATAGACATCGAAAG
	ACR-23-V295A-F	TCTATTATGATATTTATGGCATCCGACAAAATGCCATCG
	ACR-23-V295A-R	CGATGGCATTTTGTCGGATGCCATAAATATCATAATAGA
	ACR-23-S296G-F	ATTATGATATTTATGGTGGGAGACAAAATGCCATCGACG
	ACR-23-S296G-R	CGTCGATGGCATTTTGTCTCCCACCATAAATATCATAAT
	ACR-23-M299A-F	TTTATGGTGTCCGACAAAGCACCATCGACGTCTACATGT
	ACR-23-M299A-R	ACATGTAGACGTCGATGGTGCTTTGTCGGACACCATAAA
	ACR-23-P307G-F	TCGACGTCTACATGTGTTGGACTTATAGCCCTTTTCTAC
	ACR-23-P307G-R	GTAGAAAAGGGCTATAAGTCCAACACATGTAGACGTCGA
	ACR-23-I309A-F	TCTACATGTGTTCCACTTGCAGCCCTTTTCTACACACTT
	ACR-23-I309A-R	AAGTGTGTAGAAAAGGGCTGCAAGTGGAACACATGTAGA
	ACR-23-A310F-F	ACATGTGTTCCACTTATATTCCTTTTCTACACACTTATG
	ACR-23-A310F-R	CATAAGTGTGTAGAAAAGGAATATAAGTGGAACACATGT

	ACR23-Y313A-F	CCACTTATAGCCCTTTTCGCAACACTTATGATCACCATA
	ACR23-Y313A-R	TATGGTGATCATAAGTGTTGCGAAAAGGGCTATAAGTGG
	ACR23-T314A-F	CTTATAGCCCTTTTCTACGCACTTATGATCACCATAATC
	ACR23-T314A-R	GATTATGGTGATCATAAGTGCGTAGAAAAGGGCTATAAG
C. elegans strains	ACR-23-sgRNA-F	AGGATTGATACCTAAAGTCGTTTTAGAGCTAGAAATAGC
	ACR-23-sgRNA-R	GACTTTAGGTATCAATCCTCAAGACATCTCGCAATAGGA
	ACR-23-repair	CAATATTATGGAGAACTATCAAAAAGGATTGATACCTAAAGCTAGCGTCTAGTAAAGGGTCGCCAATCAATGTCACTCTTTCGTTGCAGCTTT
	dyp10-sgRNA-F	CTACCATAGGCACCACGAGGTTTTAGAGCTAGAAATAGC
	dyp10-sgRNA-R	CTCGTGGTGCCTATGGTAGCAAGACATCTCGCAATAGGA
	dyp10-repair	CACTTGAACTTCAATACGGCAAGATGAGAATGACTGGAAACCGTACCGCATGCGGTGCCTATGGTAGCGGAGCTTCACATGGCTTCAGACCAACAGCCTAT
	ACR-23-IDT-F	TGTAGATCAACGTAGATCCAGCT
	ACR-23-IDT-R	TTGCGGCTCATTCTGAAAGTTTC
	ACR-23-FSal1	CTTGCATGCCTGCAGGTCGACGTGCACCAAGAATATTTGTATTT
	ACR-23-R BamH1	TTTGGCCAATCCCGGGGATCCAAAAAGAAAATGATTTTCCGTATA
	ACR-23-Mid-F	ATTTTTTGGTTTTCCAGAAAAAAAAATATGCACAGGATCTACACATTT
	ACR-23-Mid -R	AAATGTGTAGATCCTGTGCATATTTTTTTTCTGGAAAACCAAAAAAT
