# Supplementary material for: Structural insights into the molecular effects of the anthelmintics monepantel and betaine on the Caenorhabditis elegans acetylcholine receptor ACR-23
Source: EMBO J. 2024 Jul 15;43(17):3787–806. doi: 10.1038/s44318-024-00165-7 (PMC11377560; doi:10.1038/s44318-024-00165-7)
Supplement: Supplementary file 7 — Expanded View Figures [file 44318_2024_165_MOESM7_ESM.pdf]

Expanded View Figures

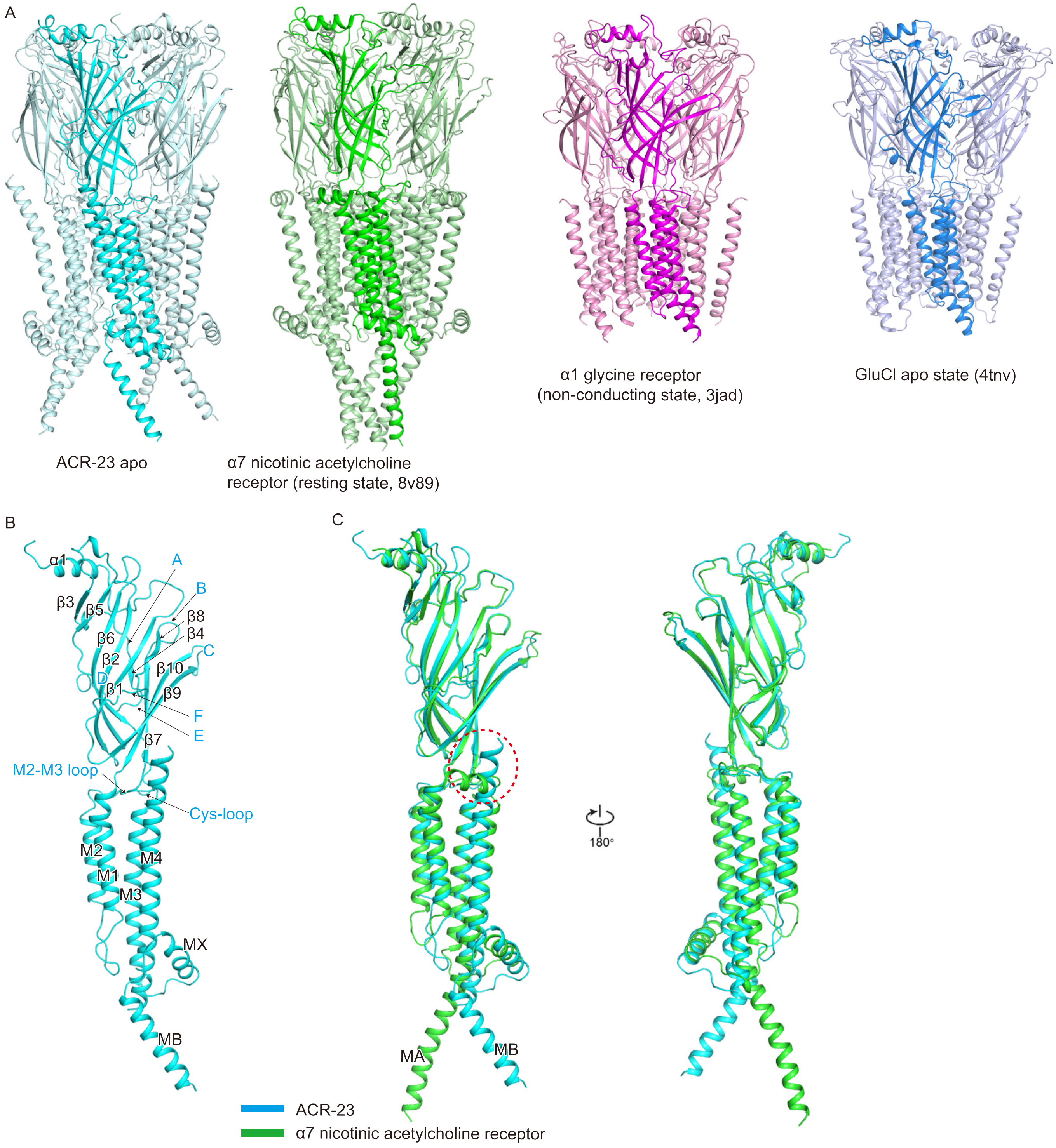

Figure EV1. Comparison of ACR-23 structure with selected Cys-loop LGICs.

(**A**) Comparison of apo structure of ACR-23 and representative Cys-loop LGICs. (**B**, **C**) Comparison of protomer structure of ACR-23 and α7 nicotinic acetylcholine receptor. In (**B**), each secondary-structure element is labeled in protomer structure of ACR-23 (shown as cartoon representations). In (**C**), superimposition of ACR-23 and α7 nicotinic acetylcholine receptor protomer structure is shown in two views, with the red circle highlighting the difference in the C-terminus of M4 (latch turn and latch helix in α7 receptor). Apo structure of ACR-23 and α7 nicotinic acetylcholine receptor structure in resting state (pdb# 7koo) are used for comparison, and are colored cyan and green respectively.

 © The Author(s)

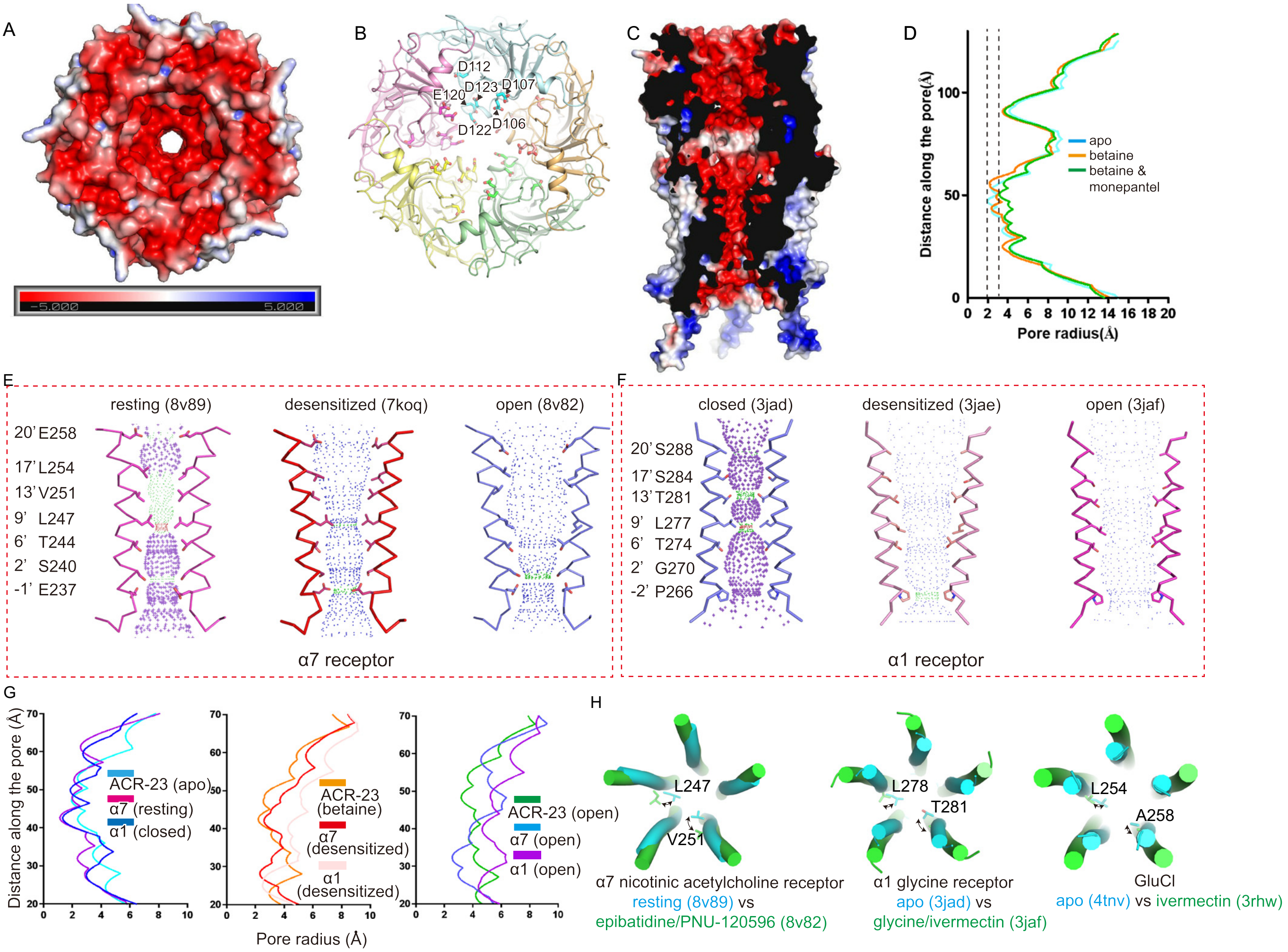

Figure EV2. Comparison of ion conduction pores of ACR-23 with selected LGICs.

(**A**) Surface electrostatic potential of ACR-23 viewed from top. (**B**) Top view of the ECD, with main chains shown as cartoons and each subunit colored differently. Rings of negatively charged residues are shown as sticks, and only one subunit is labeled for clarity. (**C**) Surface electrostatic potential along the ion permeation pathway in ACR-23, highlighting its electronegative nature. (**D**) Pore radii along the ion permeation pathway in different states of ACR-23 as calculated by the HOLE program. (**E**, **F**) Ion permeation pathway in the TMD of α7 (**E**) and α1 (**F**) receptors in closed, desensitized and open states, with main chains of M2 from two subunits and the pore lining residues shown as ribbons and sticks respectively. (**G**) Plots of pore radius for TMD of α7 and α1 receptors in closed, desensitized and open states calculated using the HOLE program, and their comparison with ACR-23 apo, betaine-bound and betaine&monepantel bound states. (**H**) Top view of superposed M2 helices from closed and open states of α7, α1, and GluCl receptors, highlighting conformational changes in the main chain and movements of the pore-lining residues. M2 helices are shown as cylinders, and side chains of key pore-lining residues are shown as sticks. For clarity, side chain of one key residue is shown on each M2 helix, with curved arrows indicating the direction of their movement.

© The Author(s)

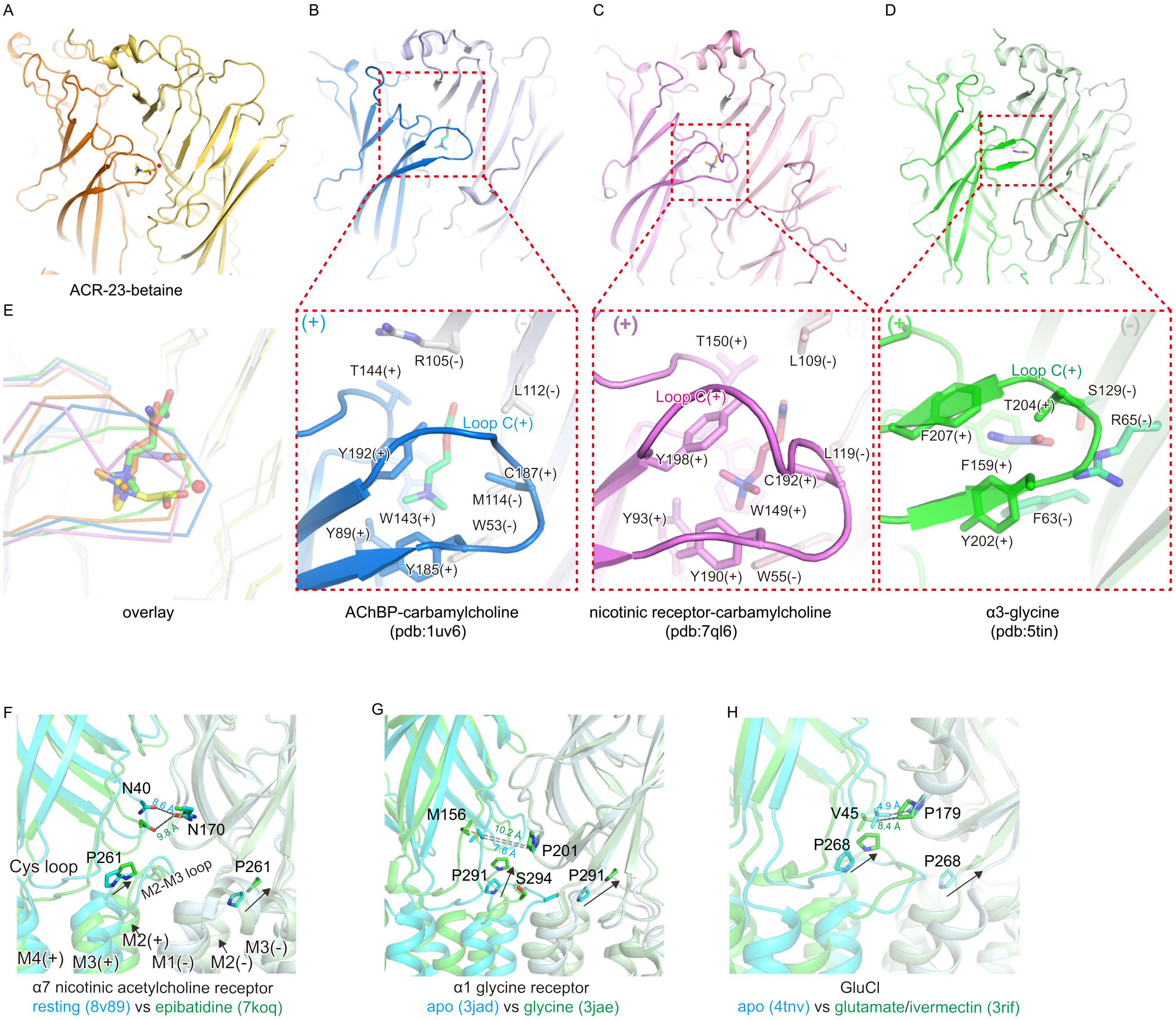

Figure EV3. Comparison of neurotransmitter binding sites among selected LGICs and conformational changes in the ECD-TMD junction induced by neurotransmitter binding.

(A–D) Two subunits of selected LGICs are shown as cartoons and colored differently, whereas neurotransmitters are shown as sticks. The name, functional state and pdb accession number of each LGIC are indicated at the bottom of each panel. In (**C**, **D**), expanded views of neurotransmitter binding sites of selected LGICs are shown in dashed boxes, with key residues shown as sticks. **(E)** Overlay of bound neurotransmitters in selected LGICs, highlighting the difference in their binding poses. **(F–H)** Overlay of closed and neurotransmitter-bound structure of selected LGICs, in which the ECD of the minus subunit of each structure is used for structure alignment. The plus and minus subunit is colored in different shades of cyan and green for the apo and neurotransmitter-bound structure, respectively. For clarity, only two adjacent subunits are shown.

 © The Author(s)

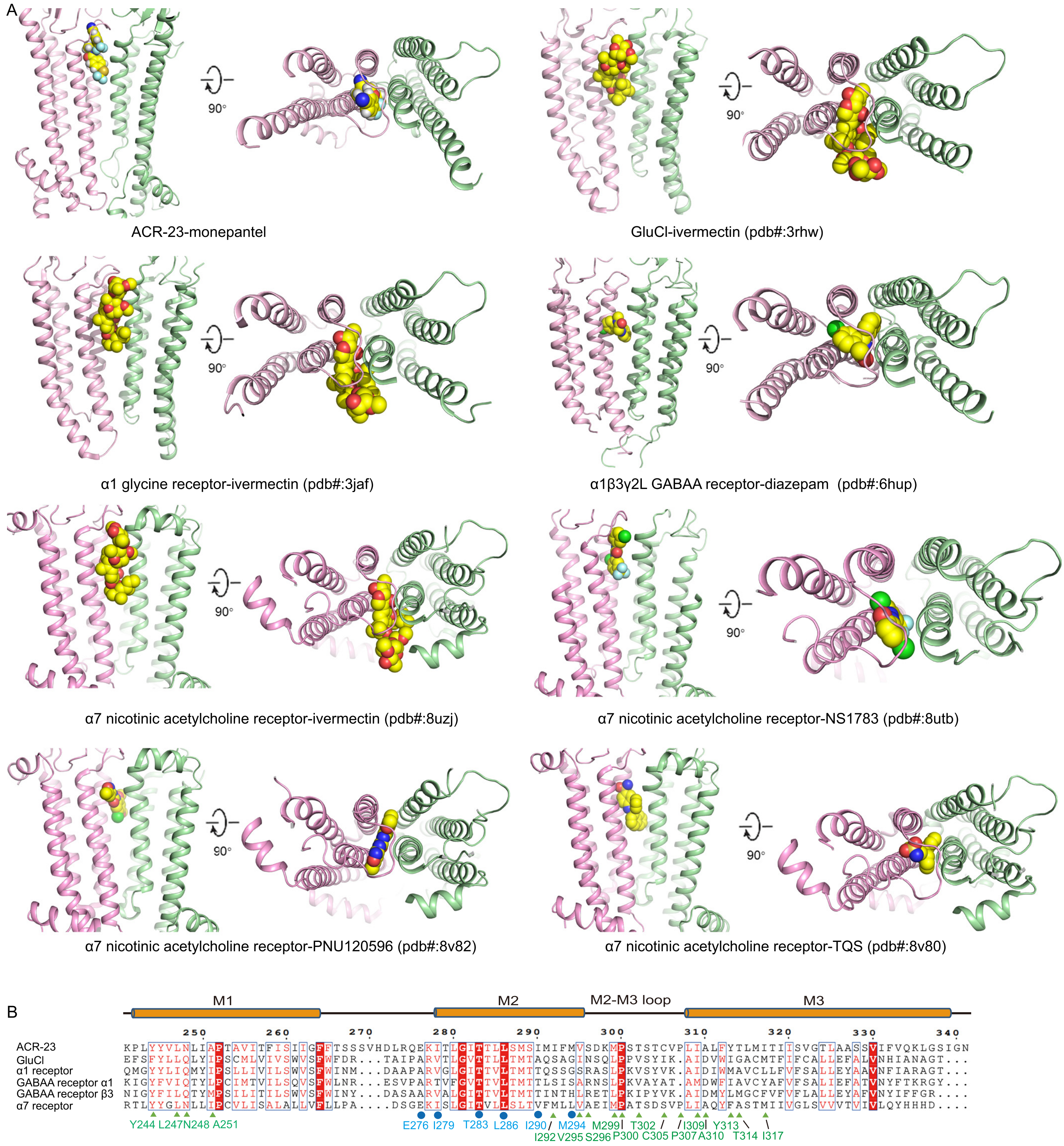

Figure EV4. Comparison of allosteric modulator binding sites among selected LGICs.

(**A**) Comparison of binding poses of allosteric modulator among selected LGICs. For each structure, two subunits are shown as cartoons and colored differently, whereas the bound allosteric modulators are shown as spheres. (**B**) Local sequence alignment spanning M1-M3 of ACR-23 and selected LGICs. Green triangles mark residues involved in monepantel binding, whereas blue circles mark pore-lining residues.

© The Author(s)

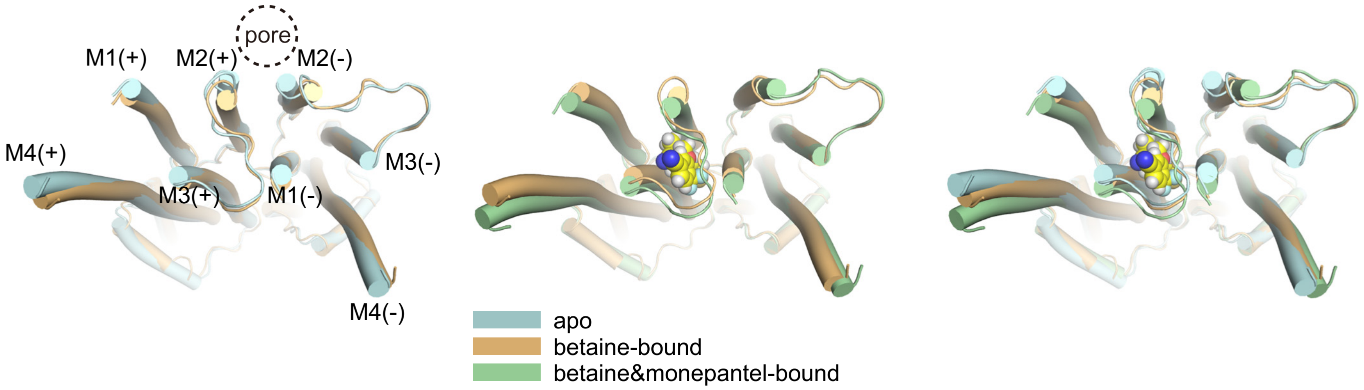

Figure EV5. Conformational changes between TMDs of ACR-23 structures in different states.

Curved arrows indicate the direction of movement. Only two subunits are shown for clarity. Main chains are shown as cartoons and bound monepantels are shown as spheres.

 © The Author(s)
